# Fundamental underpinnings of simulation education: describing a four-component instructional design approach to healthcare simulation fellowships

**DOI:** 10.1186/s41077-021-00171-3

**Published:** 2021-05-11

**Authors:** Michael J. Meguerdichian, Komal Bajaj, Katie Walker

**Affiliations:** 1 Department of Emergency Medicine, Harlem Hospital Center, NYC Health + Hospitals Simulation Center, Columbia University School of Medicine, New York, NY USA; 2 NYC Health + Hospitals Simulation Center, Bronx, NY USA; 3 Quality & Safety, NYC Health + Hospitals/Jacobi, Albert Einstein School of Medicine, Bronx, NY USA

**Keywords:** Cognitive Load Theory, Curriculum design, Faculty development, Program design, Simulation fellowship

## Abstract

Although in 2020, there are more than 120 healthcare simulation fellowships established globally, there is a paucity of literature on how to design fellowship programs most effectively, to equip graduates with the knowledge, skills, and attitudes of a competent simulation educator. Offering a systematic structure to approach simulation fellowship programmatic design may aid in better achieving program goals. In this manuscript, we present the application of the 4-component instructional design model as a blueprint to the development of Simulation Education Fellowships. We offer examples used at the NYC Health + Hospitals simulation fellowship to illustrate how the 4-component model informs fellowship program design which promotes the development of a simulation educator. This manuscript will provide a roadmap to designing curricula and assessment practices including self-reflective logbooks to focus the path toward achieving desired skills and shape future conversations around programmatic development.

## Background

Simulation fellowships have grown organically in many simulation programs throughout the world. Even with this growth, the literature offers little to understand what strategies the larger community of practice has applied to simulation fellowship program design. Several published manuscripts describe specific curricular components that are considered curricular standards during fellowship training [1–4], and one publication outlines the lack of assessment strategies to guide growth in an evolving simulation educator [5]. The American College of Surgeons and more recently, the Society for Simulation in Healthcare, offer accreditation standards for simulation fellowships to ensure there are appropriate support structures and rigor to programs. The Society for Simulation in Healthcare has developed a stellar standard of simulation educator capabilities and expertise by creating specific domains that are assessed through their Certified Healthcare Simulation Educator® (CHSE®), Certified Healthcare Simulation Educator-Advanced® (CHSE-A®), and Certified Healthcare Simulation Operations Specialist® (CHSOS®) programs. The offered domains include Professional Values and Capabilities, Healthcare and Simulation Knowledge and Principles, Educational Principles Applied to Simulation, and Simulation Resources and Environments. This evidence and these standards provide the “what” should be included in a simulation fellowship, but do not provide a guidebook on “how” to design programs focused on developing simulation educators prepared for independent practice.

In 2009, NYC Health + Hospitals (H+H), the largest municipal healthcare system in the USA, established a Simulation Center, focusing on deploying simulation to address enterprise-wide patient safety and quality priorities. As demand for the application of simulation grew across the enterprise, the program leaders agreed that more trained simulation educators were required to meet the needs of the approximate 46,000 employees of the health system.

To address the issue of simulation capacity at H+H, a simulation fellowship was established in 2011. The year-long fellowship followed an apprenticeship type model, where fellows were paired with existing simulation educators and received “on the job” training. Fellows would shadow a simulation faculty member’s course facilitation and gradually begin to facilitate more parts of the course, until they were able to teach it independently. In the apprentice-style fellowship, there were no pre-defined competencies for the fellow to meet. Fellows gradually learned elements like scenario development, debriefing, and manikin operations by observing courses. There was also inconsistency in exposure to the wide range of simulation techniques and modalities available due to the specific needs of the system limiting the breadth of activities. In successive years, it became apparent that fellows were completing the program with varied experience and expertise. Knowledge and skill gaps became evident, as fellows were asked to implement programs or facilitate courses independently within their own institutions. The Simulation Center had to think differently about how to address the performance gaps in their graduating fellows.

Most fellowships span a finite time ranging anywhere from 6 months to 2 years [6]. In that timeframe, a fellowship program is expected to cover themes such as simulation-based curriculum development, technology operations, administrative needs of a simulation program, and research strategies, using assessment in simulation and educational theory [1–4, 6]. This large body of content to be addressed, in a fixed amount of time, necessitates an overarching strategy. Cognitive Load Theory (CLT) is an instructional design approach that considers cognitive architecture and how the brain prefers to receive and process information. Most importantly, CLT proposes that memory, with regard to learning, has limitations in its packaging and storing capabilities [7–10]. Considering the large “load” to be learned by a simulation educator, CLT’s insights offer important perspectives when considering programmatic design

CLT distinguishes three main types of load: intrinsic, extraneous, and germane. Intrinsic load refers to the inherent complexity and elemental interactions of tasks and cannot be changed. Extraneous load is the working memory load experienced by learners as they interact with instructional materials. Lastly, germane load refers to the effort used by working memory to process and store information [7–10]. The literature has demonstrated over the past three decades that cognitive load strategies applied to mitigate these loads can optimize working memory’s capacity to create long-term memory, also known as schemata [11]. Some simulation literature has explored the application of CLT and how certain oversights of extraneous load and mitigation of intrinsic load may impact memory retention in simulation-based education experiences [7, 12–14]. These principles have been shown to be effective in one-off classroom learning experiences; however, adaption is required to address the inter-relatedness of ideas and skills to build expertise, in a comprehensive program over time.

Recognizing the challenge of training proficient simulation educators, with all the requisite knowledge and skills required, the Simulation Center purposefully employed the four-component instructional design (4CID) approach that draws upon the principles of CLT to provide a robust framework to re-design its fellowship program [11, 15]. The framework moves the concept of CLT from the individual classroom experience to programmatic design. This manuscript will describe the framework and provide examples of its application and fellow assessment of progression. Applying this method, NYC Health + Hospitals Simulation Center designed a fellowship training program for cohorts of 12–14 inter-disciplinary professionals over a 1-year period, meeting 1 day per week, with the goal of preparing them to be competent simulation educators at their respective institutions. To date, the program has graduated a total of 60 simulation fellows.

## Applying the 4CID approach

Merriënboer and Kirschner’s 4-component instructional design (4CID) draws upon the principles of CLT and proposes a programmatic approach to achieve not only the concepts to be taught but their inter-relatedness, also known as a “holistic approach” over time [15]. The four components include (1) structuring learning tasks, (2) offering supportive information, (3) providing procedural information, and (4) focusing part-task practice [11, 15].

According to Merriënboer and Kirschner’s approach, the instructional team must determine the skills, knowledge, and attitudes necessary to achieve competency, as a simulation educator, prior to initiating the 4CID approach. Simulation fellowship literature offers some guidance with core curricular elements [1–4]. Partnering this information with the Simulation in Healthcare Societies’ practice standards [16], the NYC Health + Hospitals simulation fellowship team derived 13 topic areas that they consider addresses the knowledge, skills, and attitudes of the developing simulation educator (Table 1).

**Table 1:** Knowledge, skills, and attitudes of a simulation educator

Topics	
Debriefing (architecture, focused facilitation, video, psychological safety)	
Interprofessional education	
Simulation operations: behind the glass	
Scenario design	
Curriculum design	
In situ simulation	
Presentation skills (virtual and live)	
Standardized patient methodology	
Procedural skills training including VR and AR	
Deliberate practice methodologies	
Simulation program management administration (i.e. structure, return on investment)	
Simulation research (i.e. journal club, research fundamentals, etc.)	
Simulation and quality improvement	

The table lists the agreed upon topic areas focused on by the Health + Hospitals simulation fellowship

The 4CID model was applied to these domains as a strategy to focus fellows’ exploration of these elements.

## Structuring learning tasks

When designing individual learning experiences for each fellow, careful attention is placed on focusing the individual’s working memory to create new schema and impact existing schema. In other words, the fellow’s ability to think, troubleshoot, and problem solve differently upon completing the program is dependent on long-term memory created through the instructional design process delivered by the program [15, 17]. As different topics require different instructional strategies, it is necessary to balance and mitigate intrinsic load and extraneous loads to avoid cognitive overload each day [7, 14, 18–20]. The program is delivered over the entire year, meeting 1 day per week, with eighteen structured educational days where the larger group gathers with a specific focus on the knowledge, skills, and attitudes of a simulation educator. Using this format, the agreed curricula, is called the Fundamental Underpinnings of Simulation Education (FUSE).

The simulation center environment and the number of interprofessional fellows set the context for the creation of dynamic learning experiences. A psychologically safe container was created at the start of the fellowship program on the first day. The lack of a psychologically safe context for learning may lead to distraction, or extraneous load, from learning the educational content provided [14, 21–23]. The commitment to respecting the learners, attending to logistical details including, scheduling, conduct, vacation and sick days, graduation requirements, clarifying objectives of the fellowship, roles, and confidentiality were all made explicit with the purpose of establishing a safe container [21]. The expectation of the program is that faculty and fellows model the behaviors made explicit on day 1 and that speaking up when psychological safety is breached is applauded. Attention to psychological safety supports honest feedback for coaching and guidance as the fellows are learning [21, 23].

The creation of immersive experiences is the cornerstone of experiential learning in simulation-based education. Merriënboer and Kirschner support learning tasks that mimic those that are reflected in real life. This can be achieved by attending to both fidelity and variability of experiences, which drives the learner toward effective translation into actual clinical practice [15]. Real-life experiences, that can be replicated in simulation, and their associated variability support implicit learning and germane processing, elements that cannot be taught in the confines of explicit instruction (i.e. lecture, group discussion, etc.). Real-life learning, in these instances, help create connections and develop schema [11, 15]. By using participants and faculty of the fellowship as learners, we recreate real-life educational experiences using simulation and foster implicit learning and schema formation. For example, when facilitating a session on difficult debriefing, faculty participate as varied phenotypes of difficult debriefing situations to allow fellows to practice mitigation strategies [24]. When fellows want to explore the effectiveness of the design of a simulation scenario they have created, classmates and faculty participate in the scenario to further explore opportunities for improvement in design.

The Fellowship Leadership purposefully allocated FUSE days in rapid sequence at the beginning of the year to lay down foundational knowledge, so that fellows have enough knowledge and skills to practice when assisting in facilitating simulation-based courses on non-FUSE days. This approach gives new fellows enough information to be competent assistant facilitators in simulation-based courses and to prevent negative transfer during teaching. From a programmatic perspective, a strategic approach was taken to help fellows navigate their learning constructively aligned with the 4-component model. By scaffolding concepts and delivering the content from simple to complex [7, 14], the intrinsic load of the concept is paired down into more manageable pieces and builds off prior knowledge. Similarly, as more expertise develops, the amount of guidance offered is decreased. This way, extraneous load is lessened by deploying instructional design in a way that avoids the expertise reversal effect. This effect recognizes that certain coaching approaches are effective with novices that may not translate as effectively as expertise increases [17, 20].

To highlight this concept, consider debriefing. Debriefing architecture is a foundational topic that is first introduced to the learner [25] through didactic and collaborative learning. The second exposure to debriefing includes complex question structures including advocacy/inquiry [26, 27] and circular questions [28, 29]. From there, additional elements are added including psychological safety during the debriefing and difficult debriefing situations, where strategies are layered on, such as normalization, attention to body language, and “sign-posting” [24]. Fellows are introduced to debriefing experiences where faculty are debriefed while maintaining certain frames to guide the fellows toward the learning objectives of applying certain strategies to mitigate psychological safety. Complexity is then increased, with the introduction of video debriefing strategies [30–32]. Each successive learning activity relies on prior schema created by the previous learning activity [7, 10, 14].

When considering fading guidance, a titration of feedback strategies is applied. Early debriefings may be coached using rapid cycle deliberate practice or pause-and-reflect [33, 34]. Rapid cycle deliberate practice is a simulation-based instructional strategy that focuses on rapid acquisition of necessary skills by repeating the skill with coached direct feedback until it is correct. Pause and reflect is a strategy where the debriefing is paused at regular intervals to allow time to reflect on what has just been said. As developing expertise emerges, feedback tools such as the Debriefing Assessment for Simulation in Healthcare (DASH) can be used. Validated assessment tools such as the DASH [35] offer more freedom to the Fellow as feedback is post-event and does not interrupt the debriefing experience. Similarly, post-event debriefing conversations are held to offer reflection and coaching. These coaching techniques are exemplary of faded guidance, as gradually the support is withdrawn. Practice in a setting of increased independence paired with variability of experience gives the developing fellow a systematic pathway to expertise. This systematic pathway provides the fellow with strategies to master the unpredictability of varied learning situations.

Establishing and maintaining psychological safety can be used as another example where scaffolding can support learning. The current approaches to psychological safety, how to set a safe container [21, 36] and maintain it [23], are first introduced through didactic teaching. Fellows begin applying the psychological safety concepts to the simulation scenario prebriefing while practicing with colleagues and receive prompt formative feedback from faculty. Faculty add complexity by intentionally creating situations that mimic safety breaches such as an upset learner during a learning experience or a content expert speaking over other faculty and learners [23]. Fellows are expected to address the breach and restore safety by applying mitigation strategies. Reflection and discussion with the faculty after the experience help reinforce these behaviors. Fellows then move their learning from simulated experiences among their classmates and faculty, to being in real debriefing situations where faculty provide faded guidance and fellows have to apply and integrate their skills with less and less faculty support as the year progresses.

Fellows participating in the program, depending on their prior schemata, exposure, and preference of learning approach, are at different levels of content mastery. As task complexity increases, there is the risk that certain learners may be overwhelmed. By choosing to teach complex learning tasks collaboratively, the processing of information can be carried over multiple working memories to lessen the load and maximize cognitive capacity [37–39]. As a result, learners can more uniformly progress toward skill acquisition together over the course of the year.

## Supportive information

Having constructs and theoretical approaches to these diverse skills is necessary for the learner to be efficient with their development. CLT suggests a mitigation strategy for intrinsic load’s task complexity by establishing theoretical frameworks for the creation of long-term memory prior to entering into a learning task [7, 14, 20]. This information is offered to the fellows through didactic, prerequisite reading, conversation, collaborative learning, and journal clubs. Taking inter-professional education (IPE) as an example, learners would be provided with multiple journal articles addressing a variety of IPE approaches for varied tasks. Once in the classroom for FUSE days, fellows are introduced to the concepts through didactic teaching and conversation. In our journal club, we weigh the benefits and pitfalls of expert approaches. Through collaborative experiences, such as learner-centered conversations, the fellows have the opportunity to explore and understand why certain elements of a interprofessional education, such as IPE in situ versus in-simulation center may link with certain outcomes in performance [40]. All these activities precede the fellow actively engaging in an interprofessional experience, which is also supported by the interprofessional nature of the fellowship. Once fellows engage in learning tasks, they have a framework from which to work and with faculty feedback can further build their long-term memory through the application of the theories.

## Procedural information

Some procedural tasks are prescriptive having a clear, stepwise approach that can become automated. These tasks have instructional approaches to create automation. Automation refers to cognitive processing that bypasses working memory. As a result, when confronted with a task, no working memory is used, and more cognitive capacity is available for execution [9]. Procedural information is used to lay down rules appropriate for carrying out specific tasks through direct feedback. Similar to Rapid Cycle Deliberate Practice, when a novice learner is performing a task that is prescriptive, errors apparent during task acquisition should be corrected immediately with direct feedback to the fellow either in private or amongst other fellows, if appropriate, to hasten their rate of proficiency acquisition [33, 41, 42]. For example, if a fellow were a novice applying the Promoting Excellence And Reflective Learning in Simulation (PEARLS) debriefing framework and skipped the “reactions” phase [43], a faculty member would stop the debriefing and instruct the fellow to correct their action by rewinding the debriefing and addressing the missed phase. Similarly, when a fellow is setting up for a simulation and powering on the manikin and associated computers, if the wrong sequence is executed by the fellow, the faculty member would stop the fellow and tactfully offer direct feedback on the correct sequence. As these tasks follow a prescribed sequence and are meant to eventually be automated, there is no reflection needed in these instances.

## Part-task practice

The fellowship targets tasks that require automation by offering part-task practice of the skills. Redundant practice of these basic skills, such as debriefing architecture, certain manikin operations, and journal review approaches, frees up more working memory to mitigate complex skills such as a difficult debriefing situation, manikin troubleshooting, and unexpected learner actions during a scenario. The task complexity would increase exponentially if there were no automation in their tasks as there are more task elements to manage. By standardizing and automating certain parts of complex tasks such as manikin set up prior to every simulation experience, or incorporating the debriefing architecture into all post-event feedback situations, the fellow is exposed multiple times per day to these structures consolidating these approaches into their long-term memory for future applications. A breakdown of the 4 components and using debriefing as a skill to contextualize the components can be found in Table 2.

**Table 2:** Application of 4-component instructional design

Component of instructional design	Application within a simulation fellowship	Examples in Debriefing
Structured learning tasks	Create real-life tasks with focused learning objectives toward desired skills acquisition Provide variability in tasks to develop systematic approaches	Use a standardized learner who offers a difficult debriefing situation Create difficult debriefing situations where strategies like “sign-posting” or normalization will de-escalate the difficult situation by creating different frames to be debriefed
Supportive information	Offer didactic covering fundamental elements of a topic Journal club	Describe debriefing architecture and the purpose of each phase Discuss journal article addressing a debriefing construct or application of debriefing
Procedural information	With skills needing to be automated, offer just-in-time feedback	During a debriefing, when an error is made skipping a phase in architecture, the debriefing is stopped and the learner is made to correct the error
Part-task practice	Focused practice on elements of a complex skill allows for chunking of information and translating it into long-term memory	Practicing the pre-brief in isolation to polish the skill prior to putting it in the larger context of the debrief

The 4-component instructional design addresses the larger program but each topic being learned and how it relates to other topics requires practical considerations and careful design consideration. The table explores these considerations around the topic of debriefing

The 4CID approach creates a framework for a program to develop its teaching strategy to address the knowledge skills and attitudes of the simulation educator. For the faculty, understanding that they are achieving these goals is important so they can appropriately adapt to learner needs. Using assessment, both self-guided and faculty-driven, helps meet that need.

## Role of reflective self-guided learning

The 4CID model supports the use of self-assessment. Some literature suggests that self-assessment is largely inaccurate, as most individuals tend to misconstrue confidence with competence [44]. Despite poor quality associated with self-assessment, this method is important to promote responsibility in individual’s learning. Doing so has the potential to stimulate germane load, the effort put forward by the working memory to package and store new information. Merriënboer and Kirschner propose that having skills for self-assessment in the future is critical for personal growth [15].

The NYC H+H simulation fellowship incorporated self-assessment in a structured way by enforcing a digital logbook. The logbook serves as a personal reference used by the learner to record both the activities performed and individual’s reflections on each activity. The learner is then asked to propose personal future activities to address any gaps, and to reinforce and refine skills. The logbook is regularly reviewed by the team and used as a guide to tailor experiences to the fellow’s self-assessed needs. Tailored experiences reinforce that the team is invested in the fellow’s growth and reinforces greater utilization of the logbook. The logbook also serves as a record to appreciate the variety of simulation experiences in which the fellow has been engaged. Review of the logbook provides guidance for the team to assign fellows to experiences that they may have missed and to ensure exposure to the widest range of health simulation activities. Without monitoring, learners may self-select out of certain activities that are required for the fellow to meet their development needs.

## Assessment

Within simulation fellowships, there are very few validated tools that are specific to fellow development and ensure acquisition of all required skills [5]. The aforementioned DASH [35] was designed as a formative assessment tool to give feedback after reviewing a video of debriefing. It has been applied in several studies in the area of post-simulation clinical debriefing [45, 46] but has never been validated as a summative tool.

To meet the gap, the NYC Health + Hospitals Simulation Center reviewed tools developed by other programs such as the milestones developed by the Accreditation Council of Graduate Medical Education (ACGME). The ACGME uses milestones as a means of offering summative assessment, across pre-determined core competencies. The tool is used by ACGME in Clinical Competency Committee Meetings for the purpose of tracking progress of trainees. The milestones use a numerical system, anchored by specific knowledge, skills, and attitudes to track the development of a competency. Although it is not plausible to include every element of the competency, the NYC Health + Hospitals team designed an assessment tool based on these milestones to meet the need of summative assessment in healthcare simulation educators. The milestones consist of 8 competencies that are listed in Table 3.

**Table 3:** NYC health + hospitals milestone competencies

Manages course delivery	
Self-reflection	
Debriefing	
Operational/technical skills	
Curriculum/scenario design	
Professional values/leadership	
Scholarly activity	
Interprofessional education	

The table offers the 8 milestones that demonstrate graduated skill acquisition of the developing fellow with an aim toward independent practice

**Fig. 1:**
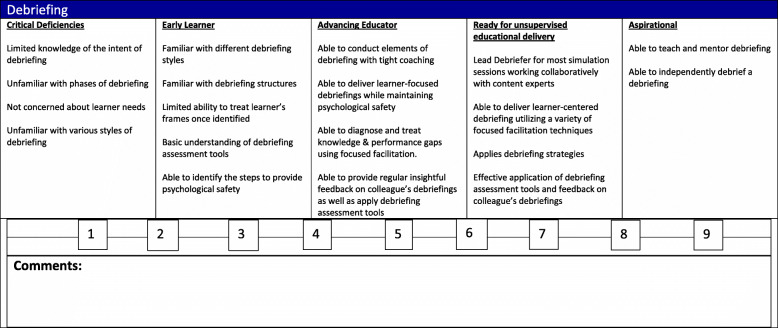
Debriefing milestone summative assessment. Similar to ACGME Milestones, the figure depicts the progression of knowledge, skills, and attitudes associated with debriefing. Each level has descriptive anchors linked with a number so that faculty can best ascribe a level of skill progression and consider mentorship or remediation to aid the fellow to ultimately achieve independent practice

An example of the milestone created for debriefing is presented in Fig. 1. The faded guidance techniques discussed earlier is similarly reflected in the summative milestones assessment as greater independence of task performance is marked by a graded increase toward independent practice and expertise. Simulation fellowship assessment is currently in the embryonic stage, and this manuscript may serve as a call to action to help address the gap in fellowship assessment as simulation fellowship training approaches continue to evolve.

Formative assessments can be offered through feedback from multiple sources (including peer feedback from other fellows and from mentors) and in a variety of settings. Regular feedback is an integral part of training for the program as a means of coaching and providing support for emerging skills. Formative assessments, such as the DASH [35], can also be pooled as means to inform summative assessment.

## Challenges of implementation

The NYC Health + Hospitals simulation fellowship program has become more robust, and the graduates since the change in the program structure have begun to establish quality simulation programs in their home facilities since the introduction of the 4CID instructional design approach. The challenges for the simulation fellowship faculty team are many. These include the markedly increased hours of curriculum development time to ensure the fellowship curriculum is embodying the 4CID model. There is also regular follow-up for each fellow by the faculty team to ensure they are recording in their logbook and working on capstone projects. Finally, each fellow receives individual feedback on their milestone progress twice throughout the fellowship to ensure they are meeting requirements and to provide opportunities to focus learning opportunities for their growth. These reviews also offer opportunities for the fellow to give the program feedback so it too can evolve and adapt. Although these challenges are not insignificant, the increased expertise of the graduating simulation fellow and return on investment from their training cannot be ignored. We look to explore this return on investment through a study that is currently underway surveying how the fellowship has impacted both the trajectory of the fellows’ career and the projects that they have undertaken to impact patient care.

## Conclusion

In this manuscript, we have applied the 4CID model as a strategy to design the NYC Health +Hospitals simulation fellowship curriculum and given examples to help guide how a program can be crafted and honed. The framework is supported by evidence-based approaches, best practices, and expert opinion. By relying on such a framework, the program is considering the cognitive load associated with simulation education knowledge, skills, and attitudes and suggested mitigation strategies. By applying such a roadmap, the goal is to develop reproducible and high-quality simulation fellowship curriculum, by which simulation fellows can create impactful simulation experiences and maybe move the dial on improved clinical outcomes. Further research into the constructs of programmatic design and development of assessment tools would be recommended to continue the maturation of simulation fellowships toward improved consistency and quality. We hope that through description of the model underpinning our program and how it has been applied, it will promote further conversation within the simulation community to shape future directions of simulation fellowships.

## Data Availability

Not applicable.
